# Soluble CLEC-2 as an Emerging Biomarker of In Vivo Platelet Activation

**DOI:** 10.3390/jcm15176651

**Published:** 2026-08-28

**Authors:** Katsue Suzuki-Inoue, Makyo Ueda, Toshiaki Shirai, Nagaharu Tsukiji, Tomoyuki Sasaki

**Affiliations:** 1 Department of Clinical and Laboratory Medicine, Faculty of Medicine, University of Yamanashi, 1110 Shimokato, Chuo, Yamanashi 409-3898, Japan; tshirai@yamanashi.ac.jp (T.S.); ntsukiji@yamanashi.ac.jp (N.T.); tomoyukis@yamanashi.ac.jp (T.S.); 2 Department of Laboratory, University of Yamanashi Hospital, 1110 Shimokato, Chuo, Yamanashi 409-3898, Japan; uedam@yamanashi.ac.jp

**Keywords:** platelets, CLEC-2, soluble CLEC-2, in vivo platelet activation marker

## Abstract

Platelet activation plays a central role in arterial thrombosis, thromboinflammation, and microvascular injury. Conventional platelet function tests evaluate platelet responsiveness to exogenous agonists ex vivo but do not directly reflect in vivo platelet activation. Although several soluble platelet-derived molecules, including platelet factor 4 (PF4), β-thromboglobulin (β-TG), soluble P-selectin, soluble CD40 ligand, glycocalicin, and soluble glycoprotein (GP) VI, have been investigated as in vivo platelet activation markers, their clinical use is limited by preanalytical instability, lack of platelet specificity, constitutive shedding, or uncertain disease specificity. Soluble C-type lectin-like receptor 2 (sCLEC-2) has recently emerged as a promising biomarker of in vivo platelet activation. CLEC-2 is expressed predominantly in platelets and megakaryocytes, and its soluble form is released from activated platelets as both a shed molecule and a microparticle-associated form. Compared with PF4 and β-TG, sCLEC-2 is less susceptible to artifactual release during routine blood collection, making it more suitable for clinical laboratory testing. Elevated sCLEC-2 levels have been reported in acute coronary syndrome, acute ischemic stroke, disseminated intravascular coagulation, thrombotic microangiopathy, antiphospholipid antibody syndrome, and coronavirus disease 2019 (COVID-19). In thrombocytopenic disorders, indices incorporating platelet count, such as the C2PAC index, sCLEC-2/D-dimer ratio, and sCLEC-2 × D-dimer/platelet count, may better reflect platelet activation and disease status than sCLEC-2 concentration alone. However, preanalytical standardization, assay harmonization, reference interval validation, and disease-specific cutoff values remain essential. This review summarizes the biological basis, assay systems, clinical evidence, and future perspectives of sCLEC-2 as an emerging laboratory marker of in vivo platelet activation.

## 1. Introduction: Unmet Need for Reliable In Vivo Platelet Activation Markers

Platelets play a pivotal role in physiological hemostasis, but they are central mediators of arterial thrombosis such as myocardial infarction and brain infarction. Assessing platelet function is necessary for monitoring the effects of antiplatelet drugs and diagnosis of platelet dysfunction. Conventional platelet function tests evaluate the responsiveness of platelets to exogenous agonists under ex vivo conditions. These include Born’s turbidimetric method and the VerifyNow^®^ system (Werfen, Barcelona, Spain), a point-of-care testing device capable of measuring whole blood. Although VerifyNow^®^ was developed for antiplatelet drug monitoring, adjusting medication based on test results has not been shown to improve prognosis [1]. UpToDate also states that platelet function tests are generally not suitable for monitoring antiplatelet therapy [2]. This may be because these tests assess the activation capacity of platelets when strong platelet agonists are added in vitro and thus do not directly reflect whether platelets are actually activated in vivo. In contrast, platelet activation in vivo can be evaluated using the release of platelet-derived proteins as an indicator. However, reliable in vivo platelet activation markers have not been established [3]. We have developed a novel in vivo marker of platelet activation: soluble C-type lectin-like receptor 2 (sCLEC-2) through our collaboration with PHC Corporation. This review introduces the development of sCLEC-2 and its clinical application. 

## 2. Mechanism of Platelet Activation and Thrombus Formation Under High-Shear Conditions In Vivo

**Figure 1:**
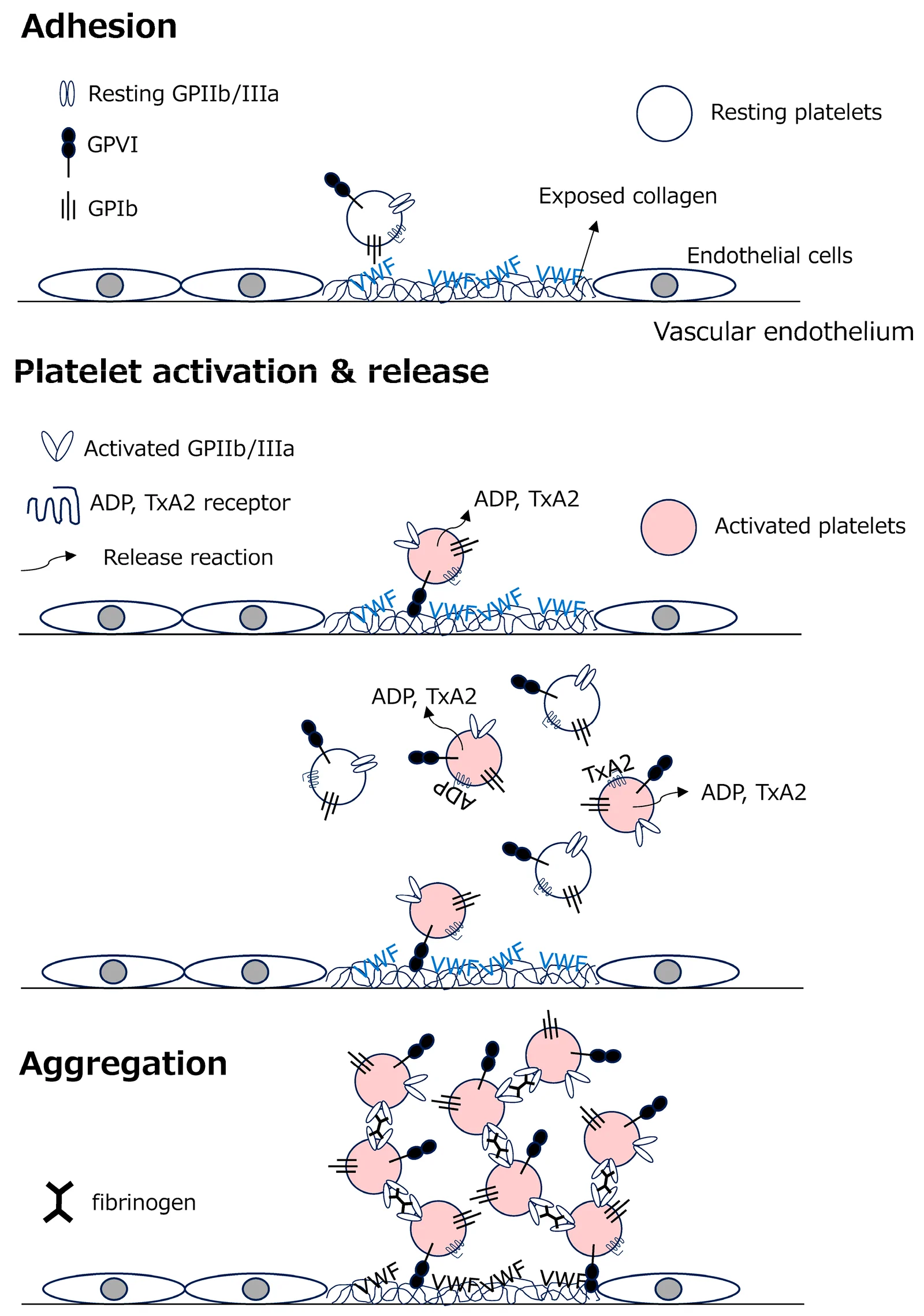
Mechanism of platelet thrombus formation in vivo. Platelet thrombus formation begins with VWF-mediated platelet adhesion to exposed subendothelial collagen through GPIb under high-shear conditions. Collagen-induced GPVI/FcRγ-chain signaling then activates GPIIb/IIIa and promotes the release of ADP and synthesis of TxA2, amplifying platelet activation. Activated GPIIb/IIIa binds fibrinogen, which bridges platelets to form a primary platelet thrombus, followed by fibrin generation and thrombus stabilization. VWF, von Willebrand factor; GPIb, glycoprotein Ib; GPVI, glycoprotein VI/Fc receptor γ-chain; GPIIb/IIIa, glycoprotein IIb/IIIa; TxA2, thromboxane A_2_.

Platelet thrombus formation proceeds through three main stages: adhesion, activation/release, and aggregation (Figure 1).

Adhesion: At sites of vascular injury or plaque rupture, plasma von Willebrand factor (VWF) binds to exposed subendothelial collagen. Under high shear conditions, such as in arteries, VWF undergoes conformational changes and binds to platelet glycoprotein Ib (GPIb), allowing platelets to tether to the vessel wall.

Platelet activation and release: Direct binding of collagen to the platelet collagen receptor GPVI/FcRγ-chain (GPVI) induces strong intracellular signaling. This activates GPIIb/IIIa, also known as integrin αIIbβ3, enabling fibrinogen binding. Activated platelets also release ADP from dense granules and synthesize thromboxane A_2_ (TxA_2_), both of which further amplify platelet activation.

Aggregation: ADP and TxA_2_ activate additional circulating platelets, leading to GPIIb/IIIa activation. Fibrinogen bridges activated platelets to form a platelet aggregate, or primary thrombus. Coagulation reactions then proceed on the activated platelet surface, generating fibrin and stabilizing the thrombus as a secondary thrombus.

## 3. In Vivo Platelet Activation Markers

**Figure 2:**
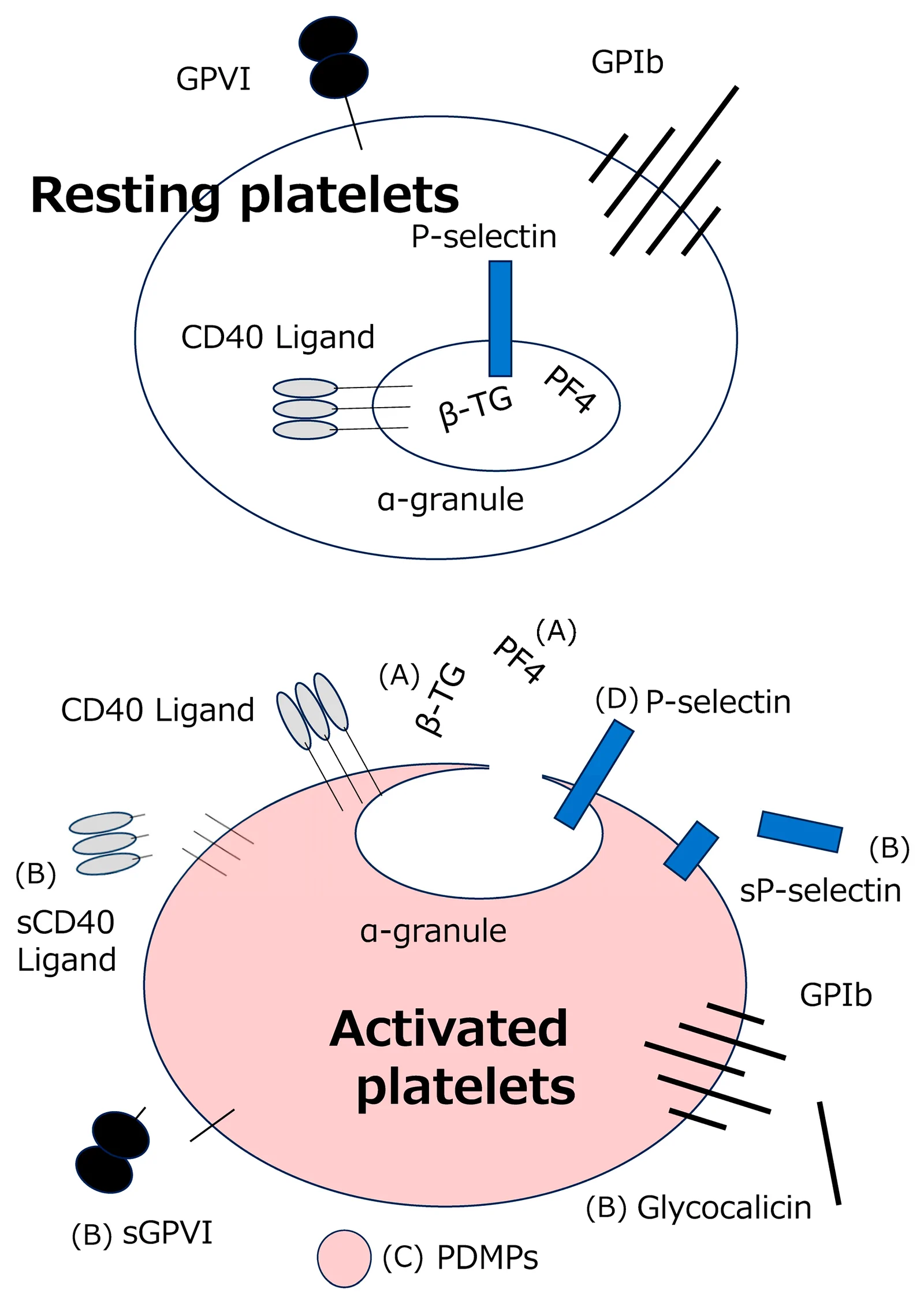
Reported in vivo platelet activation markers. (A) Platelet granule contents released upon activation, including PF4 and β-TG. (B) Soluble platelet membrane proteins released from activated platelets, including sP-selectin, sCD40L, glycocalicin (soluble GPIb), and sGPVI. (C) PDMPs, which are small membrane vesicles released from activated platelets. (D) Membrane proteins exposed on the platelet surface after activation, represented by P-selectin. PF4, platelet factor 4; β-TG, β-thromboglobulin; sP-selectin, soluble P-selectin; sCD40L, soluble CD40 ligand; sGPIb, soluble GPIb/glycocalicin; sGPVI, soluble GPVI; PDMPs, platelet-derived microparticles.

As shown in Figure 2, in vivo platelet activation markers reported to date can be broadly classified into the following categories, which are released or exposed upon platelet activation: (A) platelet granule contents, (B) released platelet membrane proteins, (C) platelet-derived microparticles (PDMPs; tiny platelet membrane vesicles released upon activation), and (D) membrane proteins exposed on the platelet surface upon platelet activation. Category A includes platelet factor 4 (PF4) and β-thromboglobulin (β-TG) found within platelet α-granules [4,5,6]. Category B includes soluble P-selectin (sP-selectin) [7], sCD40 ligand [8], sGPIb (glycocalicin) [9], and sGPVI [10]. For categories A–C, their blood concentrations are measured. Category D includes P-selectin, a membrane protein of the α-granule membrane that is expressed on the platelet surface upon activation, and its expression on platelets is measured by flow cytometry [11]. In routine clinical practice, plasma concentration measurement is more feasible and easier to standardize than flow cytometric analysis.

Of these, PF4 and β-TG are platelet-specific chemokines stored in α-granules and are released upon platelet activation. Since they are easily released by even minor physical stimulation during blood collection, special blood collection tubes and procedures are required [6]. Blood is collected without a tourniquet using a 20-gauge or larger needle and syringe, then injected into a dedicated collection tube containing an antiplatelet cocktail (CTAD tube; Citrate, Theophylline, Adenosine, Dipyridamole) and immediately chilled on ice [12]. This entire process must be completed within 2 min of blood collection. Subsequently, centrifuge the sample, transfer 0.5 mL of the supernatant to a plastic tube, and perform the assay. Since PF4 binds to the surface of vascular endothelial cells in vivo, the increase in PF4 following platelet activation in vivo is milder than the increase in β-TG. On the other hand, during platelet activation at the time of blood collection, both markers increase to a similar extent; therefore, by measuring both simultaneously, it is possible to distinguish between platelet activation in vivo and platelet activation at the time of blood collection. Although it is a good marker, the sample collection process is complex, making it unsuitable for routine clinical use; consequently, measurements are currently performed almost exclusively for research purposes, even though measurement of both markers is covered by the Japanese health insurance.

The interpretation of soluble platelet activation markers requires careful consideration of their cellular sources. P-selectin is expressed not only on platelets but also on vascular endothelial cells [13], and CD40 ligand (CD40L) is transiently induced on activated CD4-positive T cells [14] and can also be expressed by other immune cells, including mast cells and basophils [15]. Although circulating sCD40L is mainly derived from activated platelets and has been estimated to account for nearly 95% of plasma sCD40L [16,17], this molecule cannot be regarded as a strictly platelet-specific marker of activation. By contrast, GPIb and GPVI are restricted to platelets, making their soluble forms potentially more specific indicators of platelet-related processes. Nevertheless, soluble GPIb has an important limitation: GPIb undergoes constitutive shedding mediated by ADAM17, even in resting platelets [18,19]. Consequently, soluble GPIb does not necessarily reflect the magnitude of platelet activation. Before discussing the clinical significance of sGPVI and our sCLEC-2 assay, the next section outlines the biological characteristics of CLEC-2. 

## 4. Biology of CLEC-2

CLEC-2 is a platelet-activating receptor that was identified in 2006 as the receptor for the platelet-activating snake venom rhodocytin [20]. Tang et al. reported in their large-scale knockout library paper that the Clec1b/CLEC-2 protein is highly expressed on platelets and megakaryocytes, expressed at lower levels on liver Kupffer cells, but not detected on T cells, B cells, or CD31-positive endothelial cells [21]. In humans, CLEC-2/CLEC1B is expressed predominantly in platelets and megakaryocytes, with lower-level expression reported mainly in the liver [22]. Lower-level expression of CLEC-2 has also been reported in liver sinusoidal endothelial cells in humans and mice [23]. In vivo, CLEC-2 binds to the membrane protein podoplanin (PDPN) [24], which is expressed on some cancer cells [25], and induces platelet aggregation in a tyrosine kinase-dependent manner [20]. CLEC-2 is not substantially involved in hemostasis in vivo, because in normal tissues, podoplanin is expressed in lymphatic endothelium, whereas blood vascular endothelium, including arteries and capillaries, is generally negative [26]. Platelet CLEC-2 binds to PDPN on cancer cells and cancer-associated fibroblasts, promoting hematogenous metastasis and cancer-associated thrombosis (CAT) [27,28]. PDPN is also expressed in normal cells such as type I lung alveolar epithelial cells, mesothelial cells, and lymphatic endothelial cells, but not in vascular endothelial cells. Although PDPN on the lymphatic endothelial cells rarely comes into contact with platelet CLEC-2, the two interact during embryonic development to promote the blood/lymphatic vessel separation and normal lung development. In CLEC-2-deficient mouse fetuses, the separation of lymphatic and blood vessels is impaired, and lymphatic vessels filled with blood are observed [29,30]; due to pulmonary malformation, these fetuses almost always die during the transition to pulmonary respiration after birth [31]. 

## 5. Molecular Nature of sCLEC-2 and sGPVI

**Figure 3:**
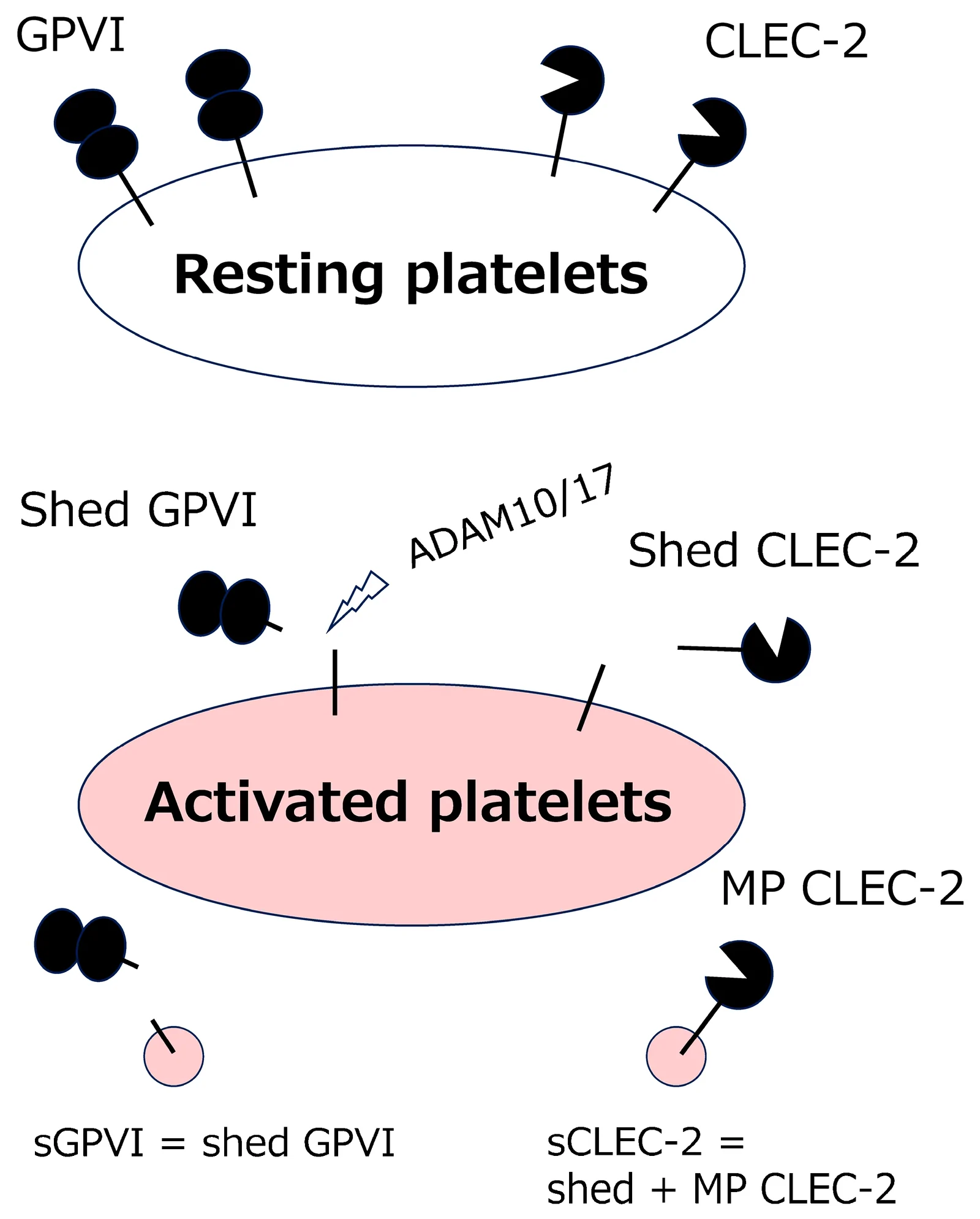
Molecular nature of sCLEC-2 and sGPVI. Upon platelet activation, GPVI is predominantly released from the platelet surface as a soluble ectodomain through proteolytic shedding. Following platelet-derived microparticle formation, intact GPVI is generally not retained on the microparticle surface; instead, the extracellular domain is lost, whereas the cytoplasmic portion may remain detectable. This pattern likely reflects the high susceptibility of GPVI to cleavage by ADAM10/17. In contrast, soluble CLEC-2 is present in two forms: a shed soluble form (Shed CLEC-2) and a microparticle-associated form (MP CLEC-2). The retention of CLEC-2 on platelet-derived microparticles may be related to its relative resistance to cleavage by ADAM10/17. Thus, unlike sGPVI, sCLEC-2 represents a heterogeneous entity composed of both a shed form and a microparticle-bound form. MP CLEC-2, microparticle-associated CLEC-2.

Receptor shedding occurs during platelet activation, probably to limit further activation [32]. Activated platelets release CLEC-2 and GPVI in different molecular forms [33] (Figure 3). 

GPVI is predominantly liberated from the platelet surface as a soluble ectodomain through proteolytic shedding [33,34]. After microparticle formation, intact GPVI is generally not retained on the microparticle surface; rather, the extracellular domain is lost, while the cytoplasmic portion may still be detectable [33]. This pattern is thought to reflect the efficient susceptibility of GPVI to cleavage by ADAM10 and ADAM17 [19,35].

In contrast, soluble CLEC-2 is present both as a shed molecule (Shed CLEC-2) and as a microparticle-associated molecule (MP CLEC-2) [33,36]. The preservation of CLEC-2 on platelet-derived microparticles may be related to its resistance to cleavage by ADAM10 and ADAM17. Therefore, sCLEC-2 should be regarded as a heterogeneous entity comprising both a soluble shed form and a microparticle-bound form. The relative contribution of each form to platelet-related pathology and its clinical relevance remain to be determined.

The basal circulating concentrations of sCLEC-2 and sGPVI differ markedly. In healthy individuals, plasma sCLEC-2 concentrations measured by ELISA are approximately 100 pg/mL, whereas plasma sGPVI concentrations are in the range of several ng/mL [10,33,37]. Thus, the circulating concentration of sGPVI is roughly 30-fold higher than that of sCLEC-2. This difference is notable because the surface copy numbers of CLEC-2 and GPVI on platelets are broadly comparable [38]. One plausible explanation is that GPVI is efficiently cleaved by ADAM10 and ADAM17, making it more susceptible to constitutive or activation-associated shedding than CLEC-2.

Clinical observations in patients undergoing coronary angiography further suggest that sCLEC-2 and sGPVI may reflect distinct aspects of platelet-related vascular pathology. In EDTA plasma samples obtained via catheter from 139 patients, both sCLEC-2 and sGPVI concentrations were elevated in patients with acute coronary syndrome compared with those with normal coronary arteries [33]. However, their patterns differed across disease categories. sCLEC-2 levels were comparable between patients with normal coronary arteries and those with stable angina pectoris, but they were significantly increased in acute coronary syndrome. In contrast, sGPVI levels were higher in stable angina pectoris than in normal coronary arteries, whereas no significant difference was observed between acute coronary syndrome and normal coronary arteries. Several mechanisms may account for this discrepancy. GPVI interacts with multiple ligands, including collagen, laminin, fibrinogen, and fibrin. During plaque disruption and thrombus formation, soluble or membrane-associated GPVI may bind to exposed subendothelial matrix components such as laminin, as well as to fibrin within the developing thrombus [39,40,41]. Such binding could attenuate the apparent increase in circulating sGPVI concentrations. Indeed, Bigalke et al. have reported lower sGPVI levels in patients with myocardial infarction than in those with stable angina pectoris [42]. In stable angina pectoris, by contrast, increased shear stress caused by coronary stenosis may promote ADAM10 activation [43], thereby favoring GPVI shedding and increasing plasma sGPVI concentrations.

CLEC-2 appears to behave differently. Because CLEC-2 has no established ligand on the vascular wall and is not efficiently cleaved by ADAM10, its soluble form may more directly reflect platelet activation associated with acute thrombotic events. These differences in ligand binding, shedding mechanisms, and vascular interactions may explain why sCLEC-2 and sGPVI show distinct profiles in coronary artery disease.

## 6. sCLEC-2 Assay Systems and Preanalytical Considerations

### 6.1. sCLEC-2 Assay Systems

To enable quantitative assessment of sCLEC-2 in plasma, our group, in collaboration with LSI Medience Corporation (Tokyo, Japan), now PHC Corporation (Tokyo, Japan), developed a monoclonal antibody against CLEC-2 and constructed a sandwich ELISA system [36], which has just been launched by Immuno-Biological Laboratories Co., Ltd. (Fujioka, Japan). This assay detects sCLEC-2 derived from both ectodomain shedding and platelet-derived microparticles. More recently, the assay has been adapted to a chemiluminescent enzyme immunoassay platform using the fully automated clinical laboratory analyzer STACIA^®^ (PHC Corporation, Tokyo, Japan), thereby improving its suitability for routine laboratory use [44]. This automated system has substantially reduced the technical barriers to clinical implementation. In March 2025, the sCLEC-2 assay kit was approved as a certified diagnostic reagent for research use, and contract testing is also available through LSI Medience in Japan.

Using the chemiluminescent enzyme immunoassay (CLEIA)-based method, the reference interval in 77 healthy individuals was established as 63.4–156.0 pg/mL [44]. The preliminary reference interval of our in-house ELISA is 42–152 pg/mL [36]. Comparison between the CLEIA and ELISA methods showed a strong correlation, with the following regression equation: CLEIA = 1.155 × ELISA + 24.21, and a correlation coefficient of r = 0.908. However, the STACIA^®^ system appears to detect the shed form of CLEC-2 predominantly, whereas its sensitivity for microparticle-associated CLEC-2 is considerably lower. The reason for this difference in detection efficiency has not yet been clarified. 

Our ELISA and CLEIA showed a strong correlation, and the reference intervals obtained with the two methods were similar. However, the molecular forms detected by the two assays are not identical. Therefore, even when overall concentrations obtained by the two methods are highly correlated, differences in the relative abundance of shed and microparticle-associated sCLEC-2 among disease states could potentially influence assay-dependent results.

It should also be noted that commercially available Human CLEC-2 ELISA kits from companies such as Sigma (Burlington, MA, USA), Abcam (Cambridge, UK), and RayBiotech (Peachtree Corners, GA, USA) have measurement ranges in the ng/mL order [45,46]. For example, the standard curve range of the kits is 1.229–300 ng/mL, and the sensitivity is 1.22 ng/mL. These analytical characteristics differ markedly from those of the PHC Corporation-based method, in which the reference interval is in the pg/mL range. This is probably because the sensitivity of the commercial ELISA is insufficient to measure the pg/mL range of sCLEC-2. In fact, the official product page for the commercial ELISA states that ‘human CLEC-2 concentration is pretty low in normal serum/plasma; it may not be detected in this assay’. Thus, values obtained using such assays cannot be directly compared with the reference intervals or clinical cutoffs established using our assays. These commercial kits have been used in a number of recent international studies. Therefore, careful attention should be paid to the assay system employed when interpreting published data on sCLEC-2.

### 6.2. Preanalytical Considerations

PF4 and β-TG are particularly sensitive to ex vivo platelet activation during venipuncture and sample processing as described in Section 3. To determine whether similarly stringent preanalytical handling is necessary for sCLEC-2 measurement, we compared samples obtained under PF4/β-TG-compatible conditions using CTAD tubes with those collected under conditions more representative of routine clinical practice. The latter included the use of 21-gauge needles, standard holders, and the commonly used vacuum blood collection tubes. As expected, PF4 and β-TG concentrations were markedly increased when blood was collected into tubes other than CTAD, indicating substantial susceptibility to collection-induced platelet activation. By contrast, sCLEC-2 concentrations were comparable among plasma samples prepared from EDTA, sodium citrate, and CTAD tubes, and showed little variation according to the blood collection procedure. These findings indicate that sCLEC-2 is less affected by preanalytical manipulation than PF4 and β-TG [36]. Accordingly, sCLEC-2 may be measured more reliably under standard clinical blood collection conditions, without the need for the highly restrictive procedures required for PF4 and β-TG.

### 6.3. Recommended Specimen Handling for sCLEC-2 Measurement

Ueda et al. examined preanalytical conditions for sCLEC-2 measurement in detail, comparing 2-mL EDTA-2K tubes, 2-mL sodium citrate tubes, and 5-mL sodium citrate tubes [47]. After centrifugation, the mean residual platelet counts in plasma prepared from all tube types were below the recommended threshold of 10 × 10^9^/L. When individual samples were assessed, all specimens collected in 2-mL EDTA and 2-mL sodium citrate tubes satisfied this criterion, whereas some samples collected in 5-mL sodium citrate tubes exceeded the recommended residual platelet count.

Additional differences were observed during sample handling. In specimens collected in 5-mL sodium citrate tubes, sCLEC-2 concentrations tended to increase after freeze–thaw cycles, and higher values were more frequently observed in the lower layer of plasma. In contrast, when samples were stored either refrigerated or frozen, sCLEC-2 levels remained largely stable for at least 28 days regardless of the tube type.

The measured values also varied according to the blood collection tube. Although the reason for this remains unclear, sCLEC-2 concentrations obtained from 5-mL sodium citrate tubes were significantly lower than those from 2-mL EDTA and 2-mL sodium citrate tubes. In addition, rare but markedly elevated values were observed in samples collected in 2-mL EDTA tubes. On the basis of these findings, Ueda et al. recommended the use of 2-mL sodium citrate tubes for sCLEC-2 measurement. Nevertheless, the type of collection tube and the details of specimen handling should be carefully considered when interpreting sCLEC-2 data.

## 7. Clinical Conditions Associated with Elevated sCLEC-2 Levels

### 7.1. Atherothrombotic Disease: Acute Coronary Syndrome and Ischemic Stroke

It has been reported that sCLEC-2 in patients with acute coronary syndrome is significantly increased compared with patients with normal coronary arteries or age-matched subjects without a previous history of atherosclerotic disease [33,48].

More extensive data have accumulated in the field of ischemic stroke, where sCLEC-2 has been investigated not only as a potential diagnostic marker but also as a prognostic indicator. In patients with acute ischemic stroke, elevated sCLEC-2 levels on admission have been shown to be independently associated with neurological deterioration and unfavorable outcomes at 90 days [49]. In another cohort of patients with acute stroke, admission sCLEC-2 concentrations were associated with subsequent mortality and vascular events during one year of follow-up [50]. These findings suggest that sCLEC-2 may reflect the intensity of platelet activation during the acute phase of cerebrovascular disease and may provide clinically relevant information beyond conventional risk assessment.

Another promising application is the differentiation of ischemic stroke subtypes. Atherothrombotic infarction and lacunar infarction are generally characterized by platelet-dominant thrombotic mechanisms and are mainly managed with antiplatelet therapy. By contrast, cardioembolic stroke is driven primarily by coagulation activation and usually requires anticoagulant therapy. Therefore, accurate discrimination between these mechanisms is clinically important. The sCLEC-2/D-dimer ratio, which integrates platelet activation reflected by sCLEC-2 and coagulation activation reflected by D-dimer, has been proposed as a useful auxiliary marker for distinguishing these stroke subtypes [51]. The composite indices using sCLEC-2 were listed in Table 1.

### 7.2. Thrombotic Diseases Associated with Thrombocytopenia

#### 7.2.1. Disseminated Intravascular Coagulation (DIC)

Because CLEC-2 is derived from platelets, sCLEC-2 concentrations show a weak positive relationship with platelet count [52,53]. This relationship requires particular attention in diseases accompanied by thrombocytopenia. In DIC, platelets are strongly activated and consumed; therefore, sCLEC-2 levels may increase despite a reduced platelet count. Conversely, the absolute sCLEC-2 concentration may underestimate the degree of platelet activation when platelet counts are markedly decreased. 

To address this limitation, the C2PAC index, calculated by normalizing sCLEC-2 to platelet count, has been proposed as a more appropriate indicator of platelet activation in thrombocytopenic conditions [52]. In one study, the C2PAC index was 0.34 in healthy individuals, 1.2 in patients with sepsis without DIC, and 2.6 in patients with sepsis complicated by DIC. The index was significantly higher in septic patients with DIC than in both healthy controls and septic patients without DIC; it was also higher in septic patients without DIC than in healthy individuals. Using a cutoff value of 1.4, the C2PAC index detected DIC with a sensitivity of 75.0% and a specificity of 76.9%. When combined with D-dimer, diagnostic performance improved further, with a sensitivity of 95.4% and a specificity of 88.4%. This improvement is biologically plausible, because DIC is fundamentally driven by coagulation activation, and D-dimer reflects secondary fibrinolysis following fibrin formation.

A related diagnostic approach incorporates D-dimer directly into the formula, using sCLEC-2 × D-dimer/platelet count [54]. In a cohort including patients with DIC, pre-DIC, and non-DIC controls with diverse underlying disorders such as infection, solid tumors, and hematological diseases, this composite index was significantly higher in the DIC group than in the non-DIC group. At a cutoff value of 17.0, the index showed high diagnostic performance for DIC/pre-DIC, with a sensitivity of 89.6%, an AUC of 0.961, and an odds ratio of 74.6. These findings suggest that combining platelet activation, coagulation activation, and platelet consumption may enhance the laboratory assessment of DIC.

#### 7.2.2. Thrombotic Microangiopathy

Thrombotic microangiopathy comprises a group of disorders characterized by platelet-rich microvascular thrombi, consumptive thrombocytopenia, and microangiopathic hemolytic anemia. In this context, sCLEC-2 may reflect platelet activation within the microcirculation. Patients with thrombotic microangiopathy have been reported to show higher sCLEC-2 levels than healthy individuals, and these levels decrease after treatment [54]. These observations suggest that sCLEC-2 may be useful for assessing disease activity and monitoring therapeutic response.

Importantly, sCLEC-2 levels appear to differ between consumptive and hypoplastic thrombocytopenia. In thrombocytopenia caused by impaired hematopoiesis, sCLEC-2 levels remain low despite a reduced platelet count. Thus, sCLEC-2 may also serve as an auxiliary marker for distinguishing platelet consumption due to thrombotic microangiopathy from decreased platelet production due to bone marrow failure.

#### 7.2.3. Antiphospholipid Antibody Syndrome

sCLEC-2 has also been evaluated in antiphospholipid antibody syndrome. In patients with antiphospholipid antibody syndrome, sCLEC-2 levels are frequently above the reference interval for healthy individuals. Elevated levels have also been observed in patients with other collagen diseases, but concentrations appear to be higher in antiphospholipid antibody syndrome than in non-APS collagen diseases [55]. These findings raise the possibility that sCLEC-2 reflects platelet activation associated with autoimmune-mediated thrombosis, although further studies are needed to clarify its disease specificity and clinical utility.

### 7.3. Coronavirus Disease 2019 (COVID-19)

Thromboinflammation and microvascular thrombosis are central features of severe COVID-19 [56,57]. In this setting, sCLEC-2 has been investigated as a marker of platelet activation associated with disease severity. In a comparison study between patients with COVID-19 and those with pneumonia caused by other infectious diseases, sCLEC-2 levels were significantly higher in the COVID-19 group [58]. This suggests that platelet activation may be more pronounced in COVID-19 than in non-COVID infectious pneumonia. The clinical relevance of this finding is supported by studies showing that sCLEC-2 levels at admission may predict the subsequent need for oxygen therapy in hospitalized patients with COVID-19 [59]. Autopsy studies have also demonstrated pulmonary microthrombi in patients with COVID-19. Taken together, these findings suggest that platelet activation may contribute to impaired pulmonary oxygenation, at least in part through microthrombus formation in the pulmonary microvasculature.

### 7.4. Other Potential Applications

Beyond cardiovascular, cerebrovascular, and thrombotic disorders, sCLEC-2 may have broader clinical applications in conditions where platelet activation contributes to disease progression. In oncology, sCLEC-2 has been investigated in relation to cancer-associated thrombosis, metastatic progression, and disease activity [60,61]. Ando et al. reported that in high-grade gliomas, especially IDH-wildtype tumors with high podoplanin expression, elevated sCLEC-2 levels and C2PAC index have been reported. A higher C2PAC index was also associated with postoperative VTE, suggesting a possible link between tumor-associated platelet activation and thrombotic risk. Platelet activation is increasingly recognized as an important component of the interaction between cancer cells, coagulation, and the vascular microenvironment; therefore, sCLEC-2 may provide additional information in selected malignancies.

Potential utility has also been reported in the prediction of prognosis after blunt head trauma [62]. Although these observations remain preliminary, they indicate that sCLEC-2 may be relevant in a variety of clinical settings characterized by platelet activation, thromboinflammation, or microvascular injury. 

Platelet reactivity varies substantially among individuals and is influenced by both acquired and genetic factors. Genetic variants in platelet signaling-related genes, including PEAR1, MRVI1, and ARHGEF3, have been investigated in association with platelet hyperaggregability and sticky platelet syndrome [63,64]. These observations raise the possibility that intrinsic platelet reactivity (platelet response ability to exogenous platelet agonists measured by aggregometry) may influence the magnitude of in vivo platelet activation (the extent to which platelets are actually activated in vivo in the patient) and consequently circulating sCLEC-2 levels under thrombotic conditions. However, whether individuals with genetically determined platelet hyperreactivity exhibit higher basal or stimulus-induced sCLEC-2 levels remains unknown and warrants further investigation.

## 8. Current Limitations and Future Directions

sCLEC-2 is currently transitioning from a research marker to a clinically applicable laboratory test in Japan; however, its use remains limited internationally. Further validation in larger, well-defined cohorts is required before sCLEC-2 can be incorporated into routine clinical practice.

Although sCLEC-2 can be measured using specimens obtained under standard clinical blood collection conditions, its measured values may be influenced by residual platelets in plasma and by the type of blood collection tube used. Therefore, standardization of preanalytical procedures, including blood collection, centrifugation, plasma separation, storage, and freeze–thaw handling, is essential. In addition, discrepancies among commercially available assay kits should be resolved, as differences in analytical sensitivity, calibration, and the molecular forms detected may lead to inconsistent results across studies.

It has been demonstrated that increased sCLEC-2 levels are associated with the presence of disease, its severity, and clinical outcomes. However, evidence that sCLEC-2 provides incremental value beyond established laboratory parameters, including platelet count, D-dimer and conventional coagulation or inflammatory markers, remains limited. In Japan, a multicenter prospective cohort study, CLECSTRO, has been conducted [65], and its findings have been submitted for publication. International collaborative studies are therefore needed to determine whether the findings obtained in Japanese cohorts can be generalized to other populations and healthcare settings. Such studies should also clarify the clinical contexts in which sCLEC-2 provides added value over established biomarkers, such as platelet count, D-dimer, and conventional coagulation or inflammatory markers.

Ultimately, the clinical utility of sCLEC-2 will depend on the establishment of standardized measurement methods, robust reference intervals, disease-specific cutoff values, and evidence that its use improves diagnostic accuracy, risk stratification, or patient management. These efforts will be essential for defining the appropriate role of sCLEC-2 in routine laboratory medicine.

## 9. Conclusions

In conclusion, sCLEC-2 is not a platelet function test but rather an in vivo platelet activation marker. It may represent a promising candidate for clinical laboratory application in Japan.

## Figures and Tables

**Table 1:** Composite indices using sCLEC-2.

Name of Composite Indices	Formula	Application
sCLEC-2/D-dimer ratio	sCLEC-2/d-dimer	Classification of Stroke Subtypes
C2PAC index	sCLEC-2/platelet count	Thrombosis with thrombocytopenia
Super formula	sCLEC-2 × D-dimer/platelet count	Diagnosis of DIC

## Data Availability

No new data were created or analyzed in this study. Data sharing is not applicable to this article.
